# Depressive symptoms and their severity in a sample with lymphedema: a case–control investigation

**DOI:** 10.3389/fpsyt.2023.1202940

**Published:** 2023-07-05

**Authors:** Ana Júlia Monteiro, Carmen de Labra, Marta Elena Losa-Iglesias, Adriano Dias, Ricardo Becerro-de-Bengoa-Vallejo, Helena Silva-Migueis, Paula Cardoso, Daniel López-López, Juan Gómez-Salgado

**Affiliations:** ^1^Research, Health, and Podiatry Group, Department of Health Sciences, Faculty of Nursing and Podiatry, Industrial Campus of Ferrol, Universidade da Coruña, Ferrol, Spain; ^2^Physiotherapy Department, Escola Superior de Saúde da Cruz Vermelha Portuguesa - Lisboa, Lisbon, Portugal; ^3^NEUROcom, Centro Interdisciplinar de Química e Bioloxía (CICA), Instituto de Investigación Biomédica de A Coruña (INIBIC), School of Nursery and Podiatry, University of A Coruña, A Coruña, Spain; ^4^Faculty of Health Sciences, Universidad Rey Juan Carlos, Alcorcon, Spain; ^5^Epidemiology – Department of Public Health and Grade Program of Public/Collective Health, Botucatu Medical School/UNESP, Botucatu, Brazil; ^6^Facultad de Enfermería, Fisioterapia y Podología, Universidad Complutense de Madrid, Madrid, Spain; ^7^Instituto Português de Oncologia de Lisboa Francisco Gentil – E.P.E. – Lisboa, Lisboa, Portugal; ^8^Department of Sociology, Social Work and Public Health, Faculty of Labour Sciences, University of Huelva, Huelva, Spain; ^9^Safety and Health Postgraduate Programme, Universidad Espíritu Santo, Guayaquil, Ecuador

**Keywords:** lower limb lymphedema, mental health, depression, somatic symptom, depressive disorder, beck depression inventory

## Abstract

**Objectives:**

Depression is a condition that can be associated with other illnesses, especially chronic illnesses. Lower limb lymphedema is a chronic, disabling condition that can affect the quality of life and be related to psychological and psychosocial factors that interfere with people’s lives. This study aims to characterize and analyze the depressive symptoms and their severity reported by people with lower limb lymphedema and compare them with a matched group without lymphedema.

**Methods:**

A case–control study was carried out (*n* = 80) with participants divided into a case group (40 people with lower limb lymphedema) and a control group (40 people without lower limb lymphedema). Both groups were anthropometrically, sociodemographically, and clinically characterized. In the case group, a characterization of lymphedema was performed. Participants completed the Beck Depression Inventory-II.

**Results:**

Individuals with lower limb lymphedema have higher BDI-II scores than the matched group without lymphedema. Somatic depressive symptoms were, in general, the most reported and the ones with the highest scores. The depressive symptoms most reported by the case group were tiredness or fatigue, loss of energy, and changes in sleeping. Tiredness or fatigue, loss of energy, and loss of interest in sex were the most severe depressive symptoms reported by individuals with lower limb lymphedema.

**Conclusion:**

Considering the apparent tendency to depression, greater attention should be given to the mental health of people with lower limb lymphedema.

## 1. Introduction

Depression is a disorder characterized by a depressed mood or loss of interest or pleasure in almost all activities, for a period equal to or longer than two weeks (1–3). In 2019, 7.2% of EU citizens reported having chronic depression, with Portugal being the country with the highest percentage of the population reporting the condition (12.2%) (4). Chronic or disabling pathological conditions are risk factors for depressive episodes (1), and depression has also been considered a risk factor for worsening comorbidities (3, 5). Depressive episodes that affect people with chronic or disabling illnesses are more likely to become chronic than those seen in healthy people. Additionally, people with depressive disorders tend to have more pain, more physical illness, and lower physical and social functioning (1).

Lymphedema is a chronic condition that is manifested by the failure of the lymphatic system and/or lymph transport, which can be primary (problems associated with lymphatic development) or secondary to another pathological condition (6–9). Lymphedema is related to negative psychological and psychosocial factors capable of interfering with treatment adherence and the quality of life of people with this condition (6, 8). These people may manifest anxiety, depression, adjustment problems, and difficulty in performing their vocational, domestic, social, and sexual activities (6). In the literature, depression has been generally associated with cancer-related lymphedema, especially breast cancer, with numerous studies showing a positive association between them (10–12). The association of depression with lower limb lymphedema is scarcer and more related to specific diagnoses, such as cancer (13), podoconiosis (14–16), and lymphatic filariasis (15, 17).

The few studies found studying the relationship between lower limb lymphedema and depression report a prevalence of at least mild depressive symptoms between 20% (17) and 86.5% (15) of their participants. As previously mentioned, these studies analyze specific causes of lymphedema, namely, tropical diseases (14–18), and were carried out in countries with socio-cultural and socio-economic characteristics very different from those of Europe. Only one of these studies analyzed the existence of depressive symptoms in people with lower limb lymphedema of other causes, with 41.9% of participants reporting mild symptoms (18). The only study found that allowed comparison with healthy people showed the existence of statistically significant differences (*p* < 0.001), with 12.6% of participants with lower limb lymphedema reporting high levels of depressive symptoms, while the healthy ones had only 0.07% (16).

Depressive symptoms seem to be present in many other chronic diseases, such as Asthma (19, 20), Chronic Obstructive Pulmonary Disease (21), Diabetes (22), Parkinson’s Disease (23), Hemophilia (24), Stroke (25), or Multiple Sclerosis (26). Lower limb lymphedema is a chronic condition that requires lifelong management and self-care that is difficult to maintain (27), capable of affecting the quality of life (28, 29), function, and appearance of people (28), which, if associated with depression, can result in increased tangible and intangible costs of this condition. On the other hand, the treatment and rehabilitation of this condition are difficult and with limited results, with the existing guidelines lacking contemporary references and better-quality scientific evidence (30).

Thus, taking into account the relationship between chronic illness and depression and its impact on the management and costs of the condition and the need to develop more integrative treatment and rehabilitation guidelines that respond to the real needs of people with lymphedema, we hypothesize that individuals with lower limb lymphedema may have higher BDI-II scores than people without the condition, showing greater prevalence and severity of depressive symptoms. Therefore, our study aims to characterize and analyze differences in BDI-II scores and the prevalence and severity of depressive symptoms reported by adult individuals with and without lower limb lymphedema from similar sociodemographic and psychosocial contexts.

## 2. Materials and methods

### 2.1. Design and sample

We performed an analytic observational case–control investigation study in people with lower limb lymphedema who participated in a foot health screening promoted by an academic clinic in the city of Lisbon (Portugal), between April 2022 and January 2023.

The study was carried out in accordance with the STROBE criteria (STROBE Statement – Checklist of items that should be included in reports of case–control studies) (31).

The sample of 80 participants was recruited through a consecutive and non-randomized sampling method. The selection of the case group was based on the following criteria: Inclusion criteria – people with lower limb lymphedema aged 18 years or older; exclusion criteria – people with cognitive disorders and/or other pathological conditions unrelated to the diagnosis of lymphedema causing depressive disorders. On the other hand, the selection of the control group included people without lower limb lymphedema aged 18 or over with similar sociodemographic characteristics and contextual origins. Other variables were included in the baseline measurements to ensure that the groups matched and that participants came from similar sociodemographic and psychosocial backgrounds.

### 2.2. Procedure

Data from this study were collected by a single senior researcher, using an Office 365 form. The researcher followed the data collection protocol imposed by the form and, subsequently, the data were exported to Excel, thus reducing transcription errors and ensuring the accuracy and integrity of data collection. The same procedure was used for the self-report of the BDI-II by the participant, without the intervention of the researcher.

Baseline measurements included demographic data such as age, weight, height, IMC (calculated by the Quetelet index) (32), professional status, profession, level of studies, and marital status; clinical data questioning the presence of other diagnoses; and lymphedema characterization data resulting from the evaluation of the onset of symptoms, the date of diagnosis, origin/triggering factor and staging.

Participants also filled out a validated outcome measure: Beck Depression Inventory-II – Portuguese version (BDI-II) (33). The BDI-II is a self-report measure developed to assess symptoms consistent with diagnostic criteria for depressive disorders in the DSM-IV in individuals aged 13 years and over, published in 1996 (33, 34). The outcome measure consists of 21 items: sadness, pessimism, past failure, loss of pleasure, guilty feeling, punishment feelings, self-dislike, self-criticalness, suicidal thoughts or wishes, crying, agitation, loss of interest, indecisiveness, worthlessness, loss of energy, changes in sleeping pattern, irritability, changes in appetite, concentration difficulty, tiredness or fatigue, and loss of interest in sex. Each item has four response options, rated from zero to three points, with zero being the absence of the symptom, one being its presence in a mild degree, two in a moderate degree, and three in a severe degree. The final score of the BDI-II is the sum of the scores of the different items and varies between 0 and 63 (33, 34). The cut-off points proposed for the interpretation of the BDI-II are: 0–13 is considered minimal range, 14–19 is mild, 20–28 is moderate, and 29–63 is severe (34). This outcome measure was cross-cultural adapted to the Portuguese context, in 2011, using two non-clinical samples, one from university students and the other from the community. These studies showed that the Portuguese version of the BDI-II has good internal consistency (0.90 < α < 0.91) and adequate convergent validity (*r* = 0.71, *p* < 0.001) with the Portuguese version of the Center of Epidemiologic Studies Depression Scale. The Portuguese version of the BDI-II also showed a two-factor structure consisting of cognitive-affective and somatic factors (33, 35). In 2023, the validation study of the Portuguese version of the BDI-II for a clinical population with cancer was published. In this study, the BDI-II showed a three-factor structure (cognitive, affective, and somatic), good internal consistency (*α* = 0.91), and good criterion validity with the MINI psychiatric interview as a discriminator (35).

### 2.3. Sample size calculation

The sample size of this case–control study was calculated for specific levels of confidence, power, and groups of equal size using the Epidat 4.2 (Program. Consellería de Sanidade, Xunta de Galicia, Spain; Organización Panamericana de la salud (OPS-OMS); Universidad CES, Colombia).

A total sample size of 80 participants (40 per group) was established taking a confidence level of 75%, a power of 0.8, an odds ratio to detect of 2.0, and an expected proportion of exposed of 50% and the controls 33.33%.

### 2.4. Ethical considerations

This study was approved by the Ethics Committee of the Portuguese Red Cross Higher Health School of Lisbon (Opinion no. 01/2022) ensuring the ethical and legal compliance of its procedures (36, 37).

### 2.5. Statistical analysis

Statistical analysis was performed using SPSS 29.0v statistical software for Windows (IBM Company, Armonk, NY, USA). A statistical significance of *p* < 0.05 and a confidence interval (CI) of 95% was established for all analyses. The normality of quantitative variables was tested using the Kolmogorov–Smirnov Test (38). All data were subject to descriptive statistics (mean, standard deviation, and range – maximum and minimum values). Differences between groups were assessed using the Student’s *t*-Test for Independent Samples (39) if the data showed a normal distribution or the Mann–Whitney U Test (40) if the distribution was non-normal. For categorical variables, frequencies and percentages were computed for comparison of groups using the Chi-Square Test (41) or Fisher’s Exact Test (42) in dichotomous variables when the 5 cases per cell criterion are not met.

## 3. Results

### 3.1. Descriptive data

A total sample of 80 individuals (12 men and 68 women), aged between 19 and 75, completed the investigation. As previously mentioned, the sample includes 40 individuals with lower limb lymphedema (case group) and 40 individuals without the condition (control group).

The sociodemographic and anthropometric characteristics of the sample are detailed in Table 1. No statistically significant differences were found between the groups for the variables: age, weight, height, BMI, BMI classification, sex, education level, and professional status. However, a statistically significant difference was found for the variable: marital relationship.

**Table 1:** Sample’s sociodemographic and anthropometric characteristics.

Descriptive Data		Total Group Mean ± SD Median ± IR Range (min-max) (*n* = 80)	Cases Mean ± SD Median ± IR Range (min-max) (*n* = 40)	Controls Mean ± SD Median ± IR Range (min-max) (*n* = 40)	*p*-value
Age (years)		51.95 ± 11.60 53 ± 14 (19–75)	51.95 ± 11.67 53 ± 14 (19–75)	51.95 ± 11.67 53 ± 14 (19–75)	1^†^
Weight (kg)		74.02 ± 21.30 69 ± 20 (42–163)	77 ± 26.82 68 ± 30.3 (43–163)	70.20 ± 13.03 70 ± 18 (42–110)	0.736^†^
Height (m)		1.64 ± 0.08 1.65 ± 0.11 (1.48–1.85)	1.63 ± 0.08 1.62 ± 0.12 (1.48–1.78)	1.64 ± 0.07 1.65 ± 0.09 (1,50-1,85)	0.312^*^
BMI (kg/m^2^)		27.45 ± 7.32 25.25 ± 7.96 (17.26–57.07)	29.11 ± 9.20 26.19 ± 10.41 (19.11–57.07)	25.78 ± 4.28 25.04 ± 6.05 (17.26–36.33)	0.392^†^
	≤ Normal	37 (%)	18 (45%)	19 (47.5%)	
	Underweight	2 (2,5%)	0 (0%)	2 (5%)	
	Normal weight	35 (43,8%)	18 (45%)	17 (42.5%)	
BMI Classification (*N* %)	Overweight	22 (27,5%)	8 (20%)	14 (35%)	0.136^‡^
	Obesity	21 (26,3%)	14(35%)	7(17.5%)	
	Obese (Class I)	12 (15%)	6 (15%)	6 (15%)	
	Obese (Class II)	4 (5%)	3 (7.5%)	1 (2.5%)	
	Obese (Class III)	5 (6,3%)	5 (12.5%)	0 (0%)	
Sex (*N* %)	Male	12 (15%)	6 (15%)	6 (15%)	1^‡^
	Female	68 (85%)	34 (85%)	34 (85%)	
Education Attainment (*N* %)	Mandatory education	12 (15%)	7 (17.5%)	5 (12.5%)	
					0.531^‡^
	≥ Mandatory education				
		63 (85%)	33 (82.5%)	35 (87.5%)	
Professional Status (*N* %)	Professionally active	64 (80%)	29 (72,5%)	35 (87,5%)	0.094^‡^
	Retired	16 (20%)	11 (27.5%)	5 (12.5%)	
Marital Relationship (*N* %)	Yes	51 (63.7%)	20 (50%)	31 (77.5%)	**0.011**^‡^
	No	29 (36.3%)	20 (50%)	9 (22.5%)	

In all analyses, *p* < 0.05 was considered statistically significant. *Student’s T-test. ^†^Mann–Whitney U test. ^‡^Chi-Squared test. SD, standard deviation; IR, interquartile range; BMI, body mass index.

The clinical characteristics of the individuals included in the case group are summarized in Table 2. In this group, there is an equal prevalence of primary and secondary lymphedema, and a similar prevalence of lymphedema committing one (47.5%) and two (52.5%) limbs. 47.5% of individuals in this group have had the condition for over 10 years and the most prevalent stage is I, with 40% of cases.

**Table 2:** Characteristics of individuals with lower limb lymphedema.

Cases *N* (%) (*n* = 40)				
Lymphedema classification	Primary	20 (50%)	Praecox	15 (37.5%)
			Tarda	5 (12.5%)
	Secondary	20 (50%)	Cancer	10 (25.0%)
			No Cancer	10 (25.0%)
Lymphedema location	Unilateral			19 (47.5%)
	Bilateral			21 (52.5%)
	<1 year			1 (2.5%)
Lymphedema duration	1–5 years			12 (30.0%)
	5–10 years			8 (20.0%)
	>10 years			19 (47.5%)
	Stage 0			5 (12.5%)
Staging of Lymphedema	Stage 1			16 (40.0%)
	Stage 2			8 (20.0%)
	Stage 3			11 (27.5%)

### 3.2. Outcome measurements

The BDI-II final score of the case group and control group is shown in Table 3, registering a statistically significant difference between both.

**Table 3:** BDI-II final score in individuals without lower limb lymphedema.

	Total Group Mean ± SD Median ± IR Range (min-max) (*n* = 80)	Cases Mean ± SD Median ± IR Range (min-max) (*n* = 40)	Controls Mean ± SD Median ± IR Range (min-max) (*n* = 40)	*p*-value^†^
BDI-II Score	11.06 ± 9,19 8.50 ± 12 (0–46)	13 ± 8.97 11.5 ± 12 (0–35)	9.13 ± 9.12 7 ± 13 (0–46)	**0.022**

*p* < 0.05 was considered statistically significant (bold). ^†^Mann–Whitney U test. SD, standard deviation; IR, interquartile range; BDI-II, Beck Depression Inventory®-II.

The results regarding the symptoms of depression assessed by the BDI-II, for both groups, are listed in Table 4 (prevalence data) and Table 5 (severity data).

**Table 4:** Prevalence of depressive symptoms in individuals with and without lower limb lymphedema.

BDI-II Items	Total Group *n* = 80	*N* (%) Cases *n* = 40	Controls *n* = 40	*p*-value
Sadness	39 (48.8%)	20 (50%)	19 (47.5%)	0.823^‡^
Pessimism	38 (47.6%)	20 (50%)	18 (45%)	0.654^‡^
Past failure	19 (23.8%)	12 (30%)	7 (17.5%)	0.189^‡^
Loss of pleasure	31 (38.9%)	20 (50%)	11 (27.5%)	**0.039** ^‡^
Guilty feelings	27 (33.8%)	16 (40%)	11 (27.5%)	0.237^‡^
Punishment feelings	18 (22.6%)	12 (30%)	6 (15%)	0.108^‡^
Self-dislike	19 (23.8%)	14 (35%)	5 (12.5%)	**0.018** ^‡^
Self-criticalness	23 (28.8%)	14 (35%)	9 (22.5%)	0.217^‡^
Suicidal thoughts	5 (6.3%)	2 (5%)	3 (7.5%)	1^¥^
Crying	27 (33.9%)	16 (40%)	11 (27.5%)	0.237^‡^
Agitation	32 (40.1%)	16 (40%)	16 (40%)	1^‡^
Loss of interest	29 (36.4%)	17 (42.5%)	12 (30%)	0.245^‡^
Indecisiveness	23 (28.8%)	14 (35%)	9 (22.5%)	0.217^‡^
Worthlessness	29 (36.3%)	20 (50%)	9 (22.5%)	**0.011** ^‡^
Loss of energy	59 (73.8%)	34 (85%)	25 (62.5%)	**0.022** ^‡^
Changes in sleeping	52 (65%)	30 (75%)	22 (55%)	0.061^‡^
Irritability	44 (55.1%)	29 (72.5%)	15 (37.5%)	**0.002** ^‡^
Changes in appetite	24 (30.1%)	10 (25%)	14 (35%)	0.329^‡^
Concentration difficulty	51 (63.9%)	28 (70%)	23 (57.5%)	0.245^‡^
Tiredness or fatigue	60 (75.1%)	36 (90%)	24 (60%)	**0.002** ^‡^
Loss of interest in sex	44 (55%)	25 (62.5%)	19 (47.5%)	0.178^‡^

In all analyses, *p* < 0.05 was considered statistically significant (bold). ^‡^Chi-Squared test. ^¥^Fisher Exact test. BDI-II, Beck Depression Inventory®-II.

**Table 5:** Severity of depressive symptoms in individuals with and without lower limb lymphedema.

BDI-II Items	Total Group Mean ± SD Range (min-max) (*n* = 80)	Cases Mean ± SD Range (min-max) (*n* = 40)	Controls Mean ± SD Range (min-max) (*n* = 40)	*p*-value^†^
Sadness	0.51 ± 0.55 (0–2)	0.50 ± 0.51 (0–1)	0.53 ± 0.60 (0–2)	1
Pessimism	0.61 ± 0.79 (0–3)	0.68 ± 0.83 (0–3)	0.55 ± 0.75 (0–3)	0.504
Past failure	0.34 ± 0.68 (0–3)	0.43 ± 0.75 (0–3)	0.25 ± 0.63 (0–3)	0.191
Loss of pleasure	0.48 ± 0.68 (0–3)	0.55 ± 0.75 (0–2)	0.40 ± 0.74 (0–3)	0.093
Guilty feelings	0.41 ± 0.63 (0–2)	0.48 ± 0.64 (0–2)	0.35 ± 0.62 (0–2)	0.284
Punishment feelings	0.31 ± 0.69 (0–3)	0.33 ± 0.53 (0–2)	0.30 ± 0.82 (0–3)	0.171
Self-dislike	0.41 ± 0.72 (0–3)	0.60 ± 0.93 (0–3)	0.23 ± 0.66 (0–3)	**0.021**
Self-criticalness	0.40 ± 0.75 (0–3)	0.48 ± 0.75 (0–3)	0.33 ± 0.69 (0–3)	0.242
Suicidal thoughts	0.06 ± 0.24 (0–1)	0.05 ± 0.22 (0–1)	0.08 ± 0.27 (0–1)	0.646
Crying	0.57 ± 0.90 (0–3)	0.62 ± 0.87 (0–3)	0.53 ± 0.93 (0–3)	0.383
Agitation	0.45 ± 0.61 (0–3)	0.43 ± 0.55 (0–2)	0.48 ± 0.68 (0–3)	0.924
Loss of interest	0.43 ± 0.63 (0–3)	0.48 ± 0.60 (0–2)	0.38 ± 0.67 (0–3)	0.283
Indecisiveness	0.36 ± 0.62 (0–2)	0.48 ± 0.72 (0–2)	0.25 ± 0.49 (0–2)	0.161
Worthlessness	0.46 ± 0.67 (0–2)	0.68 ± 0.76 (0–2)	0.25 ± 0.49 (0–2)	**0.006**
Loss of energy	0.94 ± 0.74 (0–3)	1.13 ± 0.72 (0–3)	0.75 ± 0.71 (0–3)	**0.015**
Changes in sleeping	0.78 ± 0.69 (0–3)	0.95 ± 0.75 (0–3)	0.60 ± 0.59 (0–2)	**0.032**
Irritability	0.65 ± 0.68 (0–3)	0.85 ± 0.66 (0–3)	0.45 ± 0.64 (0–2)	**0.004**
Changes in appetite	0.41 ± 0.71 (0–3)	0.38 ± 0.71 (0–2)	0.45 ± 0.71 (0–3)	0.455
Concentration difficulty	0.68 ± 0.57 (0–3)	0.70 ± 0.46 (0–1)	0.65 ± 0.66 (0–3)	0.415
Tiredness or fatigue	0.95 ± 0.69 (0–3)	1.20 ± 0.61 (0–2)	0.70 ± 0.69 (0–3)	**<0.001**
Loss of interest in sex	0.85 ± 0.94 (0–3)	1.05 ± 1.06 (0–3)	0.5 ± 0.77 (0–2)	0.103

In all analyses, *p* < 0.05 was considered statistically significant (bold). ^†^Mann–Whitney U test. SD, standard deviation; BDI-II, Beck Depression Inventory®-II.

The symptoms most referred by individuals with lower limb lymphedema were: “tiredness or fatigue” (90%), “loss of energy” (85%), and “changes in sleeping” (75%). In turn, the most frequently reported symptoms of depression in the control group were: “loss of energy” (62.5%), “tiredness or fatigue” (60%), and “concentration difficulty” (57.5%).

Statistically significant differences were found between groups regarding the prevalence of symptoms: “loss of pleasure,” “self-dislike,” “worthlessness,” “loss of energy,” “irritability,” and “tiredness or fatigue.”

Concerning the severity of depressive symptoms, it was found that the symptoms with the highest results, in the case group, were: “tiredness or fatigue,” “loss of energy” and “loss of interest in sex.” In the control group, the symptoms with the greatest impact were: “loss of energy,” “tiredness or fatigue” and “concentration difficulty.” Statistically significant differences were found between groups regarding the severity of the symptoms: “self-dislike,” “worthlessness,” “loss of energy,” “change in sleeping,” “irritability,” and “tiredness or fatigue.” “Suicidal thoughts” were the least frequent symptom and also the one with the lowest severity, in both groups.

The interpretation of the BDI-II, using severity categories, is shown in Table 6, with data from both groups. The most prevalent severity category in both groups was minimal, with 55% in the cases group and 70% in the control group. No statistically significant differences were found between the groups, taking into account the BDI-II cut-off point <13 for subclinical and clinical symptoms (34, 35, 43, 44).

**Table 6:** BDI-II interpretation – individuals with and without lower limb lymphedema.

		Total Group *N* (%) (*n* = 80)	Cases *N* (%) (*n* = 40)	Controls *N* (%) (*n* = 40)	*p*-value^‡^
Severity categories of symptoms	Lower Risk	50 (62.5%)	22 (55%)	28 (70%)	
	Minimal				
	In Risk	30 (37.5%)	18 (45%)	12 (30%)	0.166
	Mild	20 (25%)	11(27.5%)	9 (22.5%)	
	Moderate	5 (6.3%)	4 (10%)	1 (2.5%)	
	Severe	5 (6.3%)	3 (7.5%)	2 (5%)	

In all analyses, *p* < 0.05 was considered statistically significant. ^‡^Chi-Squared test. BDI-II, Beck Depression Inventory®-II.

## 4. Discussion

The objective of this investigation was to characterize and evaluate the differences between self-reported depressive symptoms, using the BDI-II, by adults with and without lower limb lymphedema. Depression has been linked to several chronic illnesses, and there is evidence that lymphedema can have negative psychosocial effects (45). As already mentioned, there is an association of depression and lymphedema of the lower limbs with cancer-related, treatment-related, or tropical causes, such as podoconiosis or lymphatic filariasis, however, as far as we know, this is the first study integrating several causes of lymphedema that compares individuals with lymphedema and the general population.

It should be noted that our sample is mostly female and that this is justified by the fact that lymphedema of the lower limbs apparently affects more women, with an extensive association of this pathology with gynecological cancer (46–48). In men, the literature is scarcer, although there is already evidence of a relationship between prostate cancer and lymphedema (49). In our sample, 25% of the individuals had lymphedema of the lower limbs secondary to cancer or its treatment. However, even though the association between lymphedema and cancer is consistent, the truth is that studies that include different causes of lower limb lymphedema continue to show samples that are mostly female (29, 50). The predominance of females in the sample may be of paramount importance in interpreting the results of this investigation, since women are more likely to develop depressive symptoms than men (51).

Another result we found is that the depressive symptoms reported with more frequency and more severity are, in both groups, somatic (52, 53). Somatic symptoms of depression are also reported more often by women than men (54, 55). Non-somatic and somatic symptoms of depression appear to be associated with impaired activity of specific serotonin and norepinephrine pathways, although the pathophysiology of depression includes many other neurobiological processes. Vegetative somatic symptoms such as “change in sleeping” and “loss of interest in sex” are also influenced by serotonin and norepinephrine. However, symptoms such as “tiredness or fatigue” may have more complex neurobiological mechanisms involving a greater number of neurotransmitters and/or motor areas of the brain and spinal pathways (55).

The BDI-II is one of the most widely used self-report outcome measures to assess the severity of depressive symptoms, with excellent psychometric properties (35). The literature suggests that the use of outcome measures to assess depressive symptoms, which include self-report of somatic symptoms, may lead to increased scores in individuals with chronic illness. This is because the somatic symptoms of depression can simultaneously be symptoms of physical illnesses (52, 56), suggesting that they should not be taken into account in your assessment (56, 57). But is this enough to justify the differences between the groups (cases and controls) for somatic symptoms such as “loss of energy,” “change in sleeping” and “tiredness or fatigue”? Fatigue is a symptom commonly reported by individuals with lymphedema (45, 58). In a study that evaluated the frequency and intensity of symptoms in individuals with lower limb lymphedema, this symptom was the most reported, with more than 75% of the sample referring to fatigue. In this same study, more than 50% of the sample also reported having difficulty sleeping (45). Fatigue is a complex symptom that can result from the pathophysiological process, for example from inflammation, or be the result of deterioration of functional status and physical condition (58), or even be a reflection of depressive processes in these patients. Although there is consensus on the high prevalence of these symptoms among individuals with lower limb lymphedema, current evidence is not sufficient to define them as a direct consequence of the disease or as a result of comorbidities such as physical deconditioning or depression, and further studies are needed to clarify these symptoms relations. It is important to note that in depression, somatic symptoms are those that seem to offer greater diagnostic accuracy, being relatively prevalent in Major Depressive Disorder (59), so outcome measures that include them should continue to be used (56, 57). Reinforcing this idea, we have the findings from the validation of the BDI-II Portuguese version for the oncology population, which clearly showed that the exclusion of somatic items does not affect the accuracy of the results (35).

Although somatic symptoms were the most mentioned and statistically different between the groups, in our investigation we also found non-somatic symptoms (“loss of pleasure,” “self-dislike” and “worthlessness”) that mostly affected the group of cases. The presence of non-somatic symptoms has been associated with subclinical stages of depression – subthreshold depression (59). However, in a study carried out with university students, the low and mild severity of symptoms was related to the low probability of developing major depressive episodes (60), which can be seen as a good indicator. Despite this, studies are needed better to define depressive symptoms in individuals with lower limb lymphedema. The studies found report only the prevalence of depression and use a different outcome measure than the one used in our investigation. Even so, both registered a higher prevalence of severe depressive symptoms than those found by us – 35% (18) and 12.6% (16). The type of lymphedema, the outcome measure used, the prevalence of genders, the severity of the data revealed, and sociocultural differences may be at the origin of these discrepancies. In the control group, we also recorded a high percentage of people at risk of depression (30%). Could other factors be responsible for the increased prevalence of depressive symptoms in both groups? Our results did not show statistically significant differences in factors that can be considered triggers for depression, such as gender, BMI, educational attainment, or professional status.

Gender differences and their relationship to depression have been previously discussed. In turn, being overweight or obese has shown a positive relationship with depression and depressive symptoms (61), regardless of sex or race (62). A study carried out with control for confounding factors and mediators relating educational attainment and depression, showed that greater educational attainment is associated with a lower risk of depression, regardless of sex (63). On the other hand, retirement has been associated with a reduced risk of depression (64), and in our study, the prevalence of retired people is low (20%). The reason for retirement may also condition the risk of depression, with evidence that early retirement (which corresponds to the difference recorded between groups) may be associated with a greater risk of depression (65).

Taking into account the results of our study, it seems reasonable to state that more studies should be carried out with the aim of better understanding the relationship between the specific symptoms of lower limb lymphedema and those of depression, with a special focus on fatigue.

However, our study has some limitations that must be considered in the interpretation and generalization of its results. The sample size should be increased in future studies, allowing for more robust conclusions and analysis of variables in different types, locations, and stages of lower limb lymphedema. The randomization of the sample should be considered in future studies, in order to reduce the probability of error in the representation of the population and the selection bias, that is, the systematic error related to the methodology of selection of the participants or with factors that influence their participation. Although a large number of confounding variables were controlled, it was certainly not possible to control for all confounding variables. In the future, controlling for other variables should be considered, taking into account the complexity of intrinsic and extrinsic factors that may predispose to depression. Memory bias can also represent a limitation, as data collection depends heavily on the participant’s ability to remember what is questioned. The BDI-II is a widely used, valid, and reliable instrument, however, it lacks validation in individuals with lower limb lymphedema and this should be considered in future studies. As strengths of our study, we must also report the age and sex-matched sample and the guarantee that the participants of both groups came from similar socio-economic and socio-demographic contexts and environments.

Nonetheless, more attention should be given to these people’s mental health (promotion and prevention), so that the psychological impact of the disease can be reduced.

## 5. Conclusion

This study suggests that individuals with lower limb lymphedema have a higher prevalence and severity of depressive symptoms than the general population. Somatic depressive symptoms were the most frequently reported. Depression should not, therefore, be undervalued in individuals with chronic diseases such as lower limb lymphedema, and it is important to introduce methods/instruments for assessing somatic and non-somatic symptoms and to introduce adequate mental health promotion and prevention strategies in addressing this condition. From this perspective, it is urgent to develop studies that help to understand the relationship between the symptoms and the burden of the problem, that facilitate the choice and adequacy of these strategies, and that provide quality scientific evidence that places mental health as a priority in treatment and rehabilitation guidelines of people with lymphedema.

## Data availability statement

The raw data supporting the conclusions of this article will be made available by the authors, without undue reservation.

## Ethics statement

The studies involving human participants were reviewed and approved by Ethics Committee of the Portuguese Red Cross Higher Health School of Lisbon (Opinion no. 01/2022). The patients/participants provided their written informed consent to participate in this study.

## Author contributions

AJM, CL, ML-I, AD, RB-d-B-V, HS-M, PC, DL-L, and JG-S: conceptualization. AJM, CL, ML-I, AD, RB-d-B-V, HS-M, PC, DL-L, and JG-S: methodology. AJM, CL, ML-I, AD, RB-d-B-V, HS-M, PC, DL-L, and JG-S: formal analysis. AJM, CL, ML-I, AD, RB-d-B-V, HS-M, PC, DL-L, and JG-S: investigation. AJM, HS-M, and PC: data curation. AJM, CL, ML-I, AD, RB-d-B-V, HS-M, PC, DL-L, and JG-S: writing—original draft preparation. CL: writing—review and editing. AJM, CL, ML-I, AD, RB-d-B-V, HS-M, PC, DL-L, and JG-S: supervision. All authors have read and agreed to the published version of the manuscript.
